# The Effect of Barbed Sutures on Complication Rates Post Colectomy: A Retrospective Case-Matched Review

**DOI:** 10.7759/cureus.29484

**Published:** 2022-09-23

**Authors:** Haven Ward, Omid Hosseini, Brianna R Taylor, kwame Opoku, Jankikeerthika Dharmarpandi, Gnanashree Dharmarpandi, Izi Obokhare

**Affiliations:** 1 School of Medicine, Texas Tech University Health Sciences Center, Lubbock, USA; 2 School of Medicine, Texas Tech University Health Sciences Center, Amarillo, USA; 3 Surgery, Texas Tech University Health Sciences Center, Amarillo, USA; 4 Internal Medicine, Texas Tech University Health Sciences Center, Amarillo, USA; 5 General Surgery, Texas Tech University Health Sciences Center, Amarillo, USA

**Keywords:** complication rates, nonbarbed suture, abdominal facial closure, colectomies, barbed suture

## Abstract

Background and objective

Colectomies are common general surgical procedures performed for a variety of gastrointestinal disorders ranging from benign to malignant. Early definitive fascial closure has been shown to improve outcomes in patients following abdominal surgery. Conventional loop sutures and their accompanying knots present several disadvantages and require technical expertise. Reducing complications has been a strong driver for innovations such as the use of barbed sutures. Barbed sutures consist of axially spaced barbed segments on each side of a midpoint at which the barbs change directions. This study is a retrospective case-matched review that evaluates the effects of barbed sutures compared to non-barbed sutures on the rates and severity of postoperative complications following colectomies for abdominal fascial closure.

Materials and methods

The study enrolled 151 patients who underwent open and minimally invasive colorectal abdominal surgeries from January 1, 2017, to November 30, 2019. Primary outcome measures included operative time, length of hospital stay, and postoperative complications compared between barbed and non-barded suture types. The sub-analysis further compared the surgical approach (open vs. robotic/laparoscopic) and incision type (Pfannenstiel vs. midline and other) between the suture types.

Results

The mean operative time for barbed sutures was 177 minutes, while it was 157 minutes for non-barbed sutures, resulting in a significant difference (p=0.0264). No significant difference was noted in postoperative complications between the groups.

Conclusions

The results of this study indicate that the use of barbed sutures in colorectal surgery does not increase the chances of postoperative infections, prolonged hospital stays, or other postoperative complications. Barbed sutures resulted in fewer class IV complications and more class I complications when compared to non-barbed sutures. Barbed sutures have proven to be beneficial in cases that require good wound approximation in high-tension areas and they eliminate the need for knots.

## Introduction

Colectomies are commonly performed general surgery procedures for a wide range of gastrointestinal disorders from benign to malignant. The open approach involves a large incision in the abdominal wall allowing for a larger visual field. However, complications following delayed open abdomen closure include organ damage, fistula, and loss of water-electrolytes and proteins, indicating the need for prompt and secure abdominal closures [1]. Early definitive fascial closure of the abdomen (<7 days) is a factor that has been shown to improve outcomes in patients following an open abdomen laparotomy [2]. The laparoscopic approach is less invasive than open but can be more difficult to suture and requires surgeons to tie knots with limited visibility. In either approach of abdominal wound closure, the fascia provides the underlying support. Hence, adopting a reliable technique that provides good tensile strength is key to preventing wound dehiscence, herniation, or other postoperative complications [3]. The ideal closure for surgical incisions is fast, easy to perform, and provides optimal wound apposition with strong but not excessive tension [4]. Conventional loop sutures and their accompanying knots present several disadvantages and require technical expertise [5]. Interrupted sutures placed along the length of an incision concentrate the tension at the individual loops, and as tissue pressure builds, the resultant inflammatory response and enzymatic tissue degranulation decrease the strength of the closure and increase the likelihood of wound dehiscence [6,7]. This increased isolated tension can also strangulate the vascular flow resulting in tissue necrosis predisposing the wound to infection and dehiscence [8,9].

Intraoperative and postoperative complications are significant causes of morbidity and mortality. They result in increased length of hospital stay, repeated surgeries, additional medical treatments, and increased costs [1,2,3,4,5]. The Clavien-Dindo (CD) system provides a standardized measure of the severity of postoperative complications following colorectal surgeries. The CD classification defines a complication as "any deviation from the expected postoperative outcome course", and measures its severity according to the level of resource use required to treat it [10]. High-grade complications require inpatient management [10]. Patients with severe complications following colorectal surgery experience a profound decrease in overall quality of life (QoL) [11].

Reducing complications has been a strong driver for innovations such as the use of barbed sutures [10]. Barbed sutures consist of axially spaced barbed segments on each side of a midpoint at which the barbs change directions. They were developed so that they anchor themselves, requiring no knots or slack management for wound closure [4]. They allow for diffuse tension along the entire length of the wound, thereby decreasing the risk of vascular strangulation [4]. These sutures do not require the surgeon to manually maintain continuous tautness after each stitch is placed, enabling the completion of a series without interruption. While barbed sutures may reduce knot-related complications, the unidirectional nature of the suture makes reversibility more difficult. It has been noted that barbed sutures have increased fibrous tissue/histological reaction formation around the barbs compared to non-barbed sutures, which may be advantageous for deep fascial repair [12].

There is a growing body of evidence regarding the safety and effectiveness of the use of barbed sutures for knotless tissue control in general and in minimally invasive gynecological procedures [13]. Barbed sutures allow for consistent tension control over the suture line and avoid the need for knots in laparoscopic procedures [14]. Isolated cases of bowel obstruction have been associated with the use of barbed sutures during laparoscopic surgery [13]. While the use of barbed sutures for wound closure in gynecological procedures have been widely investigated, there is limited data on the use of barbed suture for abdominal fascial closure. In this retrospective case-matched review, we evaluated the effects of barbed sutures compared to non-barbed sutures on the rates and severity of postoperative complications following colectomies for abdominal fascial closure.

## Materials and methods

Study design and patient selection

This was a retrospective analysis of data stored in databases at two hospitals and involved 151 patients who underwent open and minimally invasive colorectal abdominal surgeries from January 1, 2017, to November 30, 2019. Both groups were matched for similar procedures, ASA scores, and diagnoses. Patients with a history of pregnancy or previous abdominal surgery, and those undergoing simultaneous non-colorectal abdominal surgeries were excluded from this study. The information obtained from the medical records of each patient included data on age, gender, race, weight, height, BMI, smoking status, presence and type of comorbidities, ASA classification, emergent or elective surgery, surgical approach, barbed versus conventional suture, operative time, length of hospital stay, the dosage of analgesics used, postoperative infections, dehiscence, reoperative repair within 30 days, and postoperative complications according to CD grade classification. All information was encrypted before sharing them with the research team and all data were analyzed and stored using SharePoint, a Health Insurance Portability and Accountability Act (HIPAA)-compliant platform.

Study setting

This multicenter retrospective case-matched cohort study analyzed patients treated at two different hospital facilities within Northwest Texas Hospital and BSA Hospital in Amarillo, Texas. All surgeries were performed by board-certified general surgeons using similar standardized techniques. Patients were informed of the surgery procedure's risks and benefits. Patent consent was obtained, and we received an “Exemption from Formal IRB Review” from the Amarillo IRB.

Statistical analysis

Descriptive statistics are summarized as mean (standard deviation), median (interquartile range), and frequency (percentages). Primary outcome measures included operative time, length of hospital stay, and postoperative complications compared between barbed and non-barded suture types. The sub-analysis further compared surgical approaches (open vs. robotic/laparoscopic) and incision types (Pfannenstiel vs. midline and other) between the groups.

## Results

From January 1, 2017, to November 30, 2019, 151 surgeries were performed across the two medical facilities. The results comparing the demographics including gender, age at the time of surgery, sex, race, BMI, diabetic and smoking status, type of surgery, and surgical approach are shown in Table 1.

**Table 1:** Demographics of the study population *Statistically significant Categorical variables were compared using the chi-square test. Age and BMI were reported as mean and standard deviation and the groups were compared using an unpaired t-test. The two suture groups both had significantly more males than females. Surgical approach distributions were significantly different with a more open approach in both groups SD: standard deviation

Variable	Barbed suture (n=77)	Non-barbed suture (n=74)	P-value
Age, years, mean ± SD	56.65 ± 16.18	59.89 ± 16.15	0.22
Sex, n (%)	-	-	0.012*
Male	55 (71.43%)	38 (51.35%)	-
Female	22 (28.57%)	36 (48.65%)	-
Race, n (%)	-	-	0.33
Black	48 (62.34%)	53 (71.62%)	-
White	7 (9.09%)	3 (4.06%)	-
Other	22 (28.57%)	18 (24.32%)	-
BMI, mean ± SD	28.62 ± 8.71	31.38 ± 11.60	0.10
Diabetic, n (%)	11 (14.28%)	10 (13.51%)	0.89
Smoking status, n (%)	-	-	0.74
Yes	20 (25.97%)	23 (31.08%)	-
Former	18 (23.38%)	15 (20.27%)	-
No	39 (50.65%)	35 (47.30%)	-
Missing	0 (0%)	1 (1.35%)	-
Type of surgery, n (%)	-	-	0.21
Emergent	50 (64.94%)	55 (74.32%)	-
Elective	27 (35.06%)	19 (25.68%)	-
Surgical approach, n (%)	-	-	0.041*
Open	50 (64.94%)	45 (60.81%)	-
Laparoscopic	14 (18.18%)	24 (32.43%)	-
Robotic	13 (16.88%)	5 (6.76%)	-

**Figure 1:**
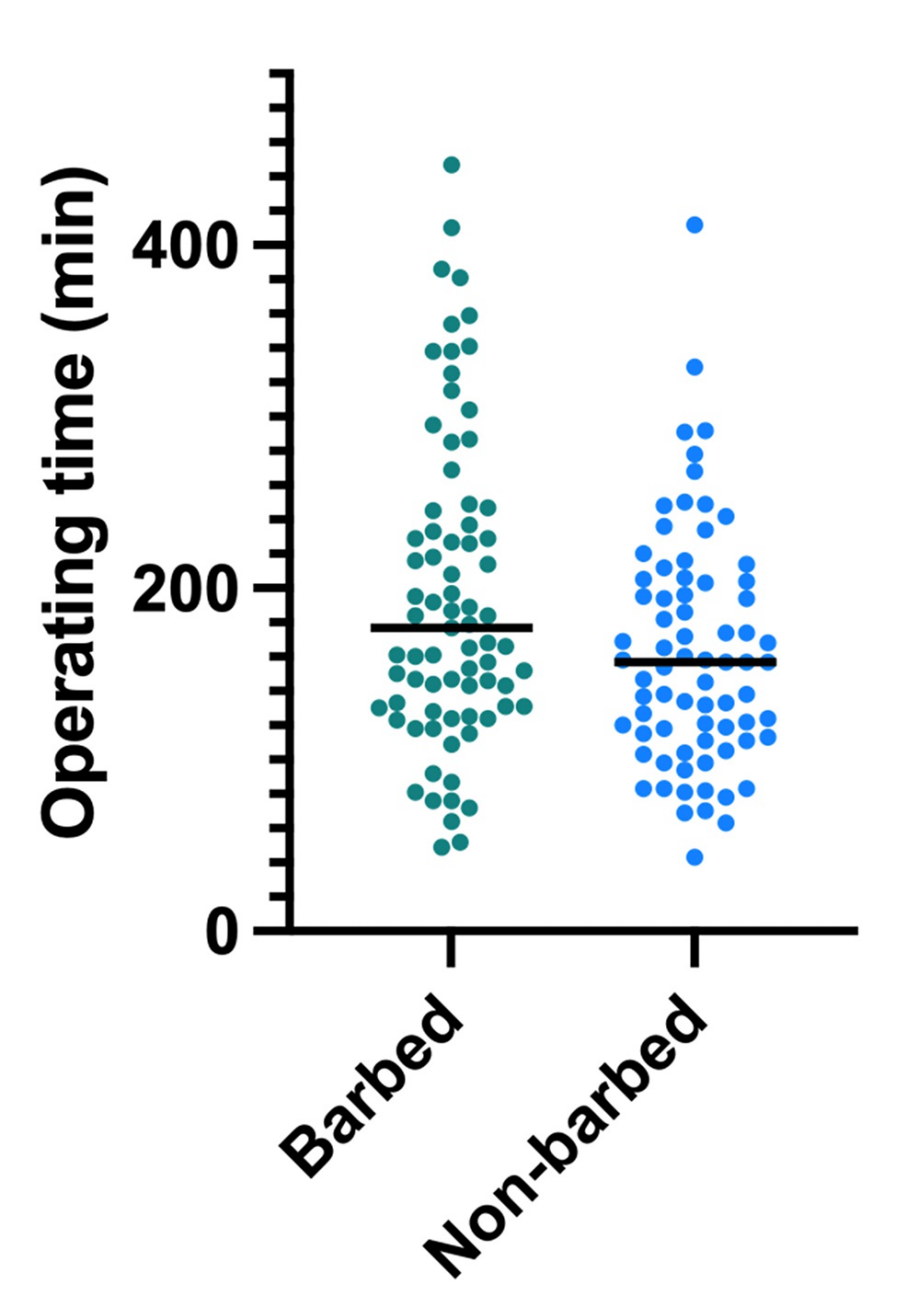
Operating time for hernia repair Individual operating times are plotted along with their respective median. The groups were compared using the Mann-Whitney test. The median operating time for the barbed suture group was 177 minutes compared to 157 minutes for the non-barbed suture group (p=0.0264)

The two demographic aspects that differed significantly were sex and surgical approach with p-values of 0.012 and 0.041, respectively. The mean operative time for barbed sutures was 177 minutes, while it was 157 minutes for non-barbed sutures. The Mann-Whitney test was used to compare the mean operative times between the two procedures and showed a significant difference (p=0.0264) (Figure 1).

**Figure 2:**
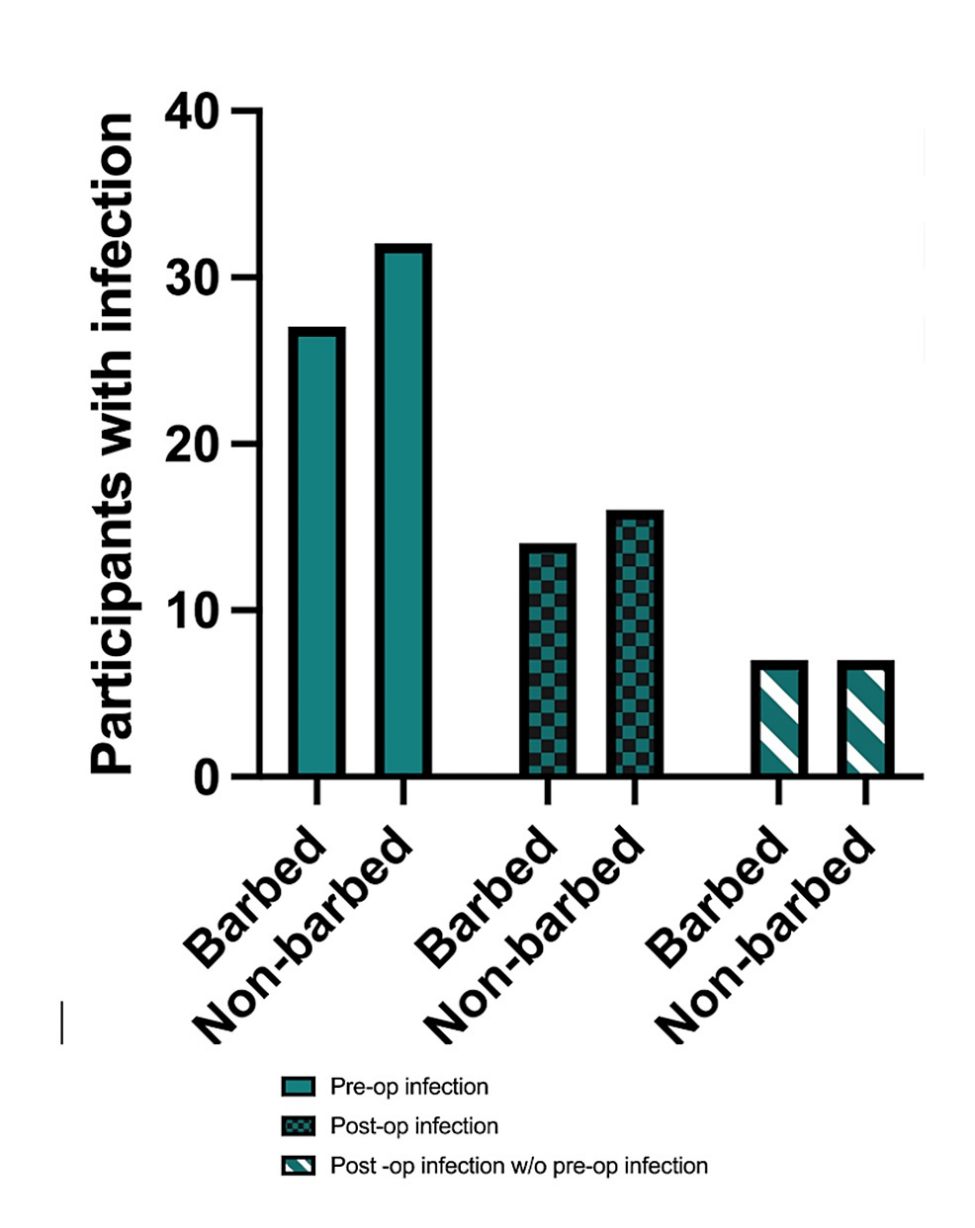
Pre and postoperative rates of infection Counts are presented as total participants with preoperative infections, postoperative infections, and postoperative infections without preceding preoperative infection. Rates were compared using a chi-square test between groups and no statistically significant difference was found (preop infection: p=0.30, postop infection: p=0.60, postop w/o preop infection: p=0.94)

Preoperative and postoperative infection rates were presented as follows: total participants with preoperative infections, postoperative infections, and postoperative infections without preceding preoperative infections. Rates were compared using chi-square tests between groups and no statistically significant difference was found (Figure 2).

**Figure 3:**
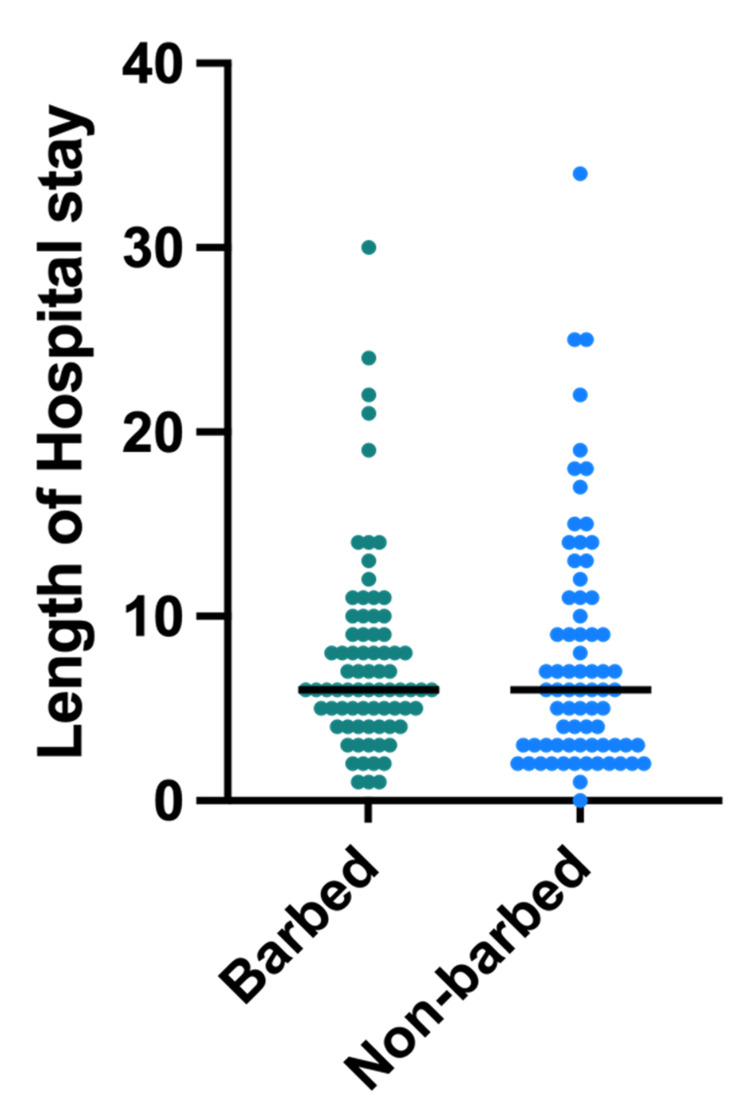
Length of hospital stay The median hospital stay for both barbed and non-barbed suture groups was six days. There was no statistically significant difference between the two groups (Mann-Whitney, p=0.46)

Results comparing the median hospital stay can be found in Figure 3.

The mean hospital stay for both barbed and non-barbed sutures was six days with no statistically significant difference (p=0.46). The rates of dehiscence and postoperative repair were compared using chi-square tests and were found to have no statistically significant differences between the two groups (Table 2).

**Table 2:** Rates of dehiscence and postoperative repair Rates of dehiscence and postoperative repair were compared using the chi-square test. There were no statistically significant differences between barbed and non-barbed groups

Variable	Barbed suture (n=77)	Non-barbed suture (n=74)	P-value
Dehiscence	1	2	0.54
Postoperative repair within 30 days			0.17
Yes, due to dehiscence	0	2	
Yes, due to another surgical reason	14	19	
No or unrelated to surgery	63	53	

**Figure 4:**
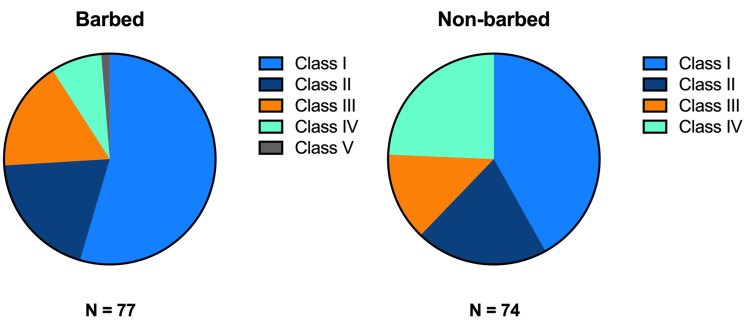
Postoperative complications represented by Clavien-Dindo grade The barbed suture group had more class I complications compared to the non-barbed. The non-barbed group had more class IV complications, though both of these differences were not statistically significant (chi-square test, p=0.06)

Postoperative complications were represented using the CD grading system. Although there was no significant difference between the groups, the barbed suture group had more class I complications, while the non-barbed suture group had increased numbers of class IV complications (Figure 4). 

A sub-analysis further comparing the two groups based on surgical approach (open vs. laparoscopic/robotic) and incision site (midline vs. Pfannenstiel) was performed using chi-square tests. The Mann-Whitney test was utilized to calculate the differences in medians between operative time and postoperative complications. When comparing the surgical approach, operative time and postoperative complications differed significantly (p=0.0003 and p=0.046, respectively) (Table 3).

**Table 3:** Comparison of groups by surgical approach *Statistically significant The chi-square test was used to assess differences between groups. Mann-Whitney test was utilized to calculate differences in medians between operative time and hospital duration. Postop complications in the open approach and operative time in the laparoscopic/robotic approach differed significantly

Variable	Barbed suture (n=77)	Non-barbed suture (n=74)	P-value
Sample size of surgical approach – open	50	45	0.60
Operative time	155	165	0.96
Infection	-	-	-
Preoperative	20	21	0.51
Postoperative	12	15	0.31
Postoperative infection w/o preop infection	6	6	0.85
Length of hospital stay	8	8	0.44
Dehiscence	1	1	0.94
Reoperative repair within 30 days	-	-	0.18
Yes, due to dehiscence	0	1	-
Yes, due to another surgical reason	12	17	-
No or unrelated to surgery	38	27	-
Postoperative complications (Clavien-Dindo grade)	-	-	0.046*
I	20	14	-
II	12	7	-
III	12	8	-
IV	5	16	-
V	1	0	-
Sample size of surgical approach – laparoscopic/robotic	27	29	0.60
Operative time	216	137	0.0003*
Infection	-	-	-
Preoperative	7	11	0.34
Postoperative	2	1	0.51
Postoperative infection w/o preop infection	1	0	0.29
Length of hospital stay	5	3	0.09
Dehiscence	0	1	0.33
Reoperative repair within 30 days	-	-	0.62
Yes, due to dehiscence	0	1	-
Yes, due to another surgical reason	2	2	-
No or unrelated to surgery	25	26	-
Postoperative complications (Clavien-Dindo grade)	-	-	0.31
I	22	17	-
II	3	8	-
III	1	2	-
IV	1	2	-
V	0	0	-

There were no statistically significant differences when comparing incisional sites (Table 4). 

**Table 4:** Comparison of groups by incision site The chi-squared test was used to assess differences between groups. Mann-Whitney test was utilized to calculate differences in medians between operative time and hospital duration. There was no statistically significant difference between the two groups across all variables

Variable	Barbed suture (n=77)	Non-barbed suture (n=74)	P-value
Sample size of surgical approach – midline/other	67	67	0.49
Operative time	165	147	0.07
Infection	-	-	-
Preoperative	26	29	0.60
Postoperative	13	15	0.67
Postoperative infection w/o preop infection	6	6	1
Length of hospital stay	6	6	0.84
Dehiscence	1	2	0.56
Reoperative repair within 30 days	-	-	0.18
Yes, due to dehiscence	0	2	-
Yes, due to another surgical reason	13	18	-
No or unrelated to surgery	54	46	-
Postoperative complications (Clavien-Dindo grade)	-	-	0.09
I	36	27	-
II	11	13	-
III	13	10	-
IV	6	17	-
V	1	0	-
Sample size of surgical approach – Pfannesteil	10	7	0.49
Operative time	278	169	0.27
Infection	-	-	-
Preoperative	1	3	0.12
Postoperative	1	1	0.76
Postoperative infection w/o preop infection	1	1	0.76
Length of hospital stay	5.5	3	0.10
Dehiscence	0	0	-
Reoperative repair within 30 days	-	-	0.36
Yes, due to dehiscence	0	0	-
Yes, due to another surgical reason	1	0	-
No or unrelated to surgery	9	8	-
Postoperative complications (Clavien-Dindo grade)	-	-	0.45
I	6	4	-
II	4	2	-
III	0	0	-
IV	0	1	-
V	0	0	-

## Discussion

The results of this study indicate that the use of barbed sutures in colorectal surgery does not increase the chances of postoperative infections, prolonged hospital stays, or postoperative complications. A study by Ruiz-Tover et al. investigated the effect on surgical site infection and evisceration using barbed sutures for fascial closure in patients undergoing emergent surgery. Their study resulted in a lower risk of evisceration with barbed sutures when compared with loop sutures (p=0.019) and no significant difference in surgical site infection (p=0.65) [15]. While our study did not result in increased rates of adverse effects, it did result in significantly longer operative times with a mean increase of 20 minutes when using barbed sutures. This could be due to the limited experience our surgeons had with barbed sutures and the time of the study.

Our study showed no significant difference in barbed versus non-barbed sutures when comparing midline to Pfannenstiel incisional types. A study performed by Murtha et al. investigated the use of barbed sutures for the closure of Pfannenstiel incision during nonemergent cesarean delivery. No adverse events were reported, and the study did not result in increased rates of surgical site infections (p=1.0), or dehiscence (p=1.0) [4].

Based on a significant outcome of this study, the use of barbed sutures results in fewer class IV complications and more class I complications when compared to the use of non-barbed sutures. A study done by Bosma et al. demonstrated that patients with minor complications (class I-II) had similar outcomes in QoL to patients without complications [11]. Those with severe complications (class III, IV, V) had a more pronounced decrease in overall QoL, QoL-physical, and QoL-psychological domains in the first six postoperative weeks [11]. A study investigating the correlation between CD complication grade and severity identified a strong correlation between CD grade and increased direct medical cost during the first 90 days after surgery [10]. Data regarding costs specific to our areas of interest are scarce; however, a New Zealand study showed that colorectal cancer resection surgery and inpatient stay for patients without complications cost $14,697, while those with complications cost $28,485. In the group with complications, costs were further broken down by complication class (I-V). Patients with minor complications could expect to pay $17,786 (class I) to $24,446 (class II). Those with major complications accrued higher bills, as follows: $37,009/$40,071, class IIIa/b; $34,846, class IV; $77,760, class V [16].

It should be noted that our cohort was relatively small, and there were limitations with respect to operative times as they varied between each surgeon’s experience. Given the retrospective nature of our study, other limitations include inherent recall bias and variations in patient charting. The study analysis was computed using the available data, and not all data points were obtained. Furthermore, our study does not touch on the effects of the learning curve. Future studies should limit their study to either only laparoscopic or open approach to reduce confounding variables.

## Conclusions

Several methods for abdominal wall closure have been studied throughout the history of colorectal surgery. The need for diffuse and adequate tension control along the entire length of an incision has led to the invention of the barbed suture. Barbed sutures have proven to be beneficial in cases that require good wound approximation in high-tension areas and they eliminate the need for knots. This study showed no significant difference in postoperative infections or complication rates for fascial closure following colorectal surgery. Therefore, the use of barbed sutures can be considered a safe and effective method in colorectal surgery for fascial closure. The present study prospectively assessed the correlation between CD grade and suture type. The results may also be used to educate our patients more thoroughly about the possible impact the complications could have on their QoL in terms of using barbed versus non-barbed sutures.
